# Employers' Subscriptions and Compensation Cases

**Published:** 1914-08-01

**Authors:** Herbert J. Dafforne

**Affiliations:** Secretary. Ancoats Hospital, Manchester


### THE EDITOR'S LETTER-BOX.

### Employers' Subscriptions and Compensation Cases.

*To the Editor of* The Hospital.

Sir,—Your interview with Mr. Roden Orde is very interesting, and brings to my mind further problems with which hospital managers are faced, especially in respect of employers' subscriptions and compensation cases.

It is always a "thorn in the flesh" to me to think that hospitals have to treat compensation cases whose employers have them insured against accident in insurance companies. These companies, in many instances, do not subscribe one penny towards the cost of the treatment of these patients, and yet it depends very largely on the skill and treatment they receive at the hands of the voluntary hospitals as to the amount of compensation which they have to pay out. Should a man meet with severe injuries to his arm, but be so carefully treated and nursed that amputation would not be necessary, these companies would not have to pay so much, and one would think that it is in their best interests as a business concern that they should be large subscribers to the voluntary hospitals who save them paying out so much money in the course of one year.

If these compensation cases were treated by a private doctor, the doctor's account would, of course, be presented as part of the expense to which the patient had been put through the accident, but when these cases are brought to the hospital, they are admitted, treated for a few weeks free of cost, go out, claim, and often get, £50 to £100 compensation, and do not pay the hospital one penny.

Then I have in mind the large employer of labour who subscribes £5 5s. per annum, but the cost of the treatment of the accident cases received from his firm exceeds this amount in six months. When this fact is pointed out, the reply is that all his accident cases are insured in a certain insurance company, and we ought to claim on them.

I should be glad to learn if there are any hospitals who have been able to obtain an adequate payment from insurance companies for the treatment of "compensation cases."—Yours faithfully,

Herbert J. Dafforne, Secretary.

Ancoats Hospital, Manchester.

